# Impact of three miRNA signature as potential diagnostic marker for triple negative breast cancer patients

**DOI:** 10.1038/s41598-023-48896-7

**Published:** 2023-12-08

**Authors:** Vivek Kumar, Mansi Gautam, Amit Chaudhary, Bipin Chaurasia

**Affiliations:** 1 Molecular Genetic Laboratory, Department of Pathology/Lab Medicine, All India Institute of Medical Sciences, Academic Building, Phulwarisharif, Patna, 801507 India; 2 Department of Medical Biotechnology, Galgotias University, Greater Noida, Uttar Pradesh 201306 India; 3 Metallurgical Engineering & Materials Science, Indian Institute of Technology Bombay, Powai, 400076 India; 4 Department of Neurosurgery, Neurosurgery Clinic, Birgunj, Nepal

**Keywords:** Biotechnology, Cancer

## Abstract

Breast cancer is a highly aggressive type of cancer and has several subtypes, including triple-negative breast cancer (TNBC), which accounts for 25% of morbidity related to breast cancer. miRNAs are small non-coding RNA molecules that regulate 60% of human genes. Dysregulated expression of miRNA in liquid biopsy of TNBC patients has the potential as a minimally invasive diagnostic biomarker. The Association of miRNA with TNBC was evaluated using in-silico analysis. Highly enriched miRNAs were selected for functional analysis to evaluate the role of miRNA in the progression of TNBC. The qRT-PCR-based expression analysis of miRNA was performed in 190 serum samples (139 TNBC and 51 healthy). Revealed the elevated expression of miRNA-155 and miRNA-21 in TNBC compared to control samples (*P* < 0.0001), while miRNA-205 was significantly downregulated in TNBC (*P* < 0.0001). The combined diagnostic value of the miRNA-205, miRNA-155 and miRNA-21 in cohort-I, cohort-II, and cohort-III was AUC of 96.1% (*P* < 0.0001), 94.9% (*P* < 0.0001), and 97.1% (*P* < 0.0001), respectively. Our study revealed that dysregulated expression of miRNA could be used as an independent indicator for discriminating TNBC from healthy patients. In addition, the combined predictive value of miRNA-205 + miRNA − 155 + miRNA-21 has higher AUC, sensitivity, and specificity in the diagnosis of TNBC in all three cohorts.

## Introduction

Breast cancer is a heterogenous and common malignancy among women worldwide. According to World Health Organization (WHO), the incidence rate of breast cancer was 2.3 million, while 6,85,000 worldwide morbidities were reported in 2021. Similarly, according to data from National Cancer Registry Program, India-2021, breast cancer has been the second leading cause of cancer, with the highest morbidity rate in the Indian scenario. Moreover, taking into account population growth, 3.2 million per year of breast cancer cases will be rendered globally by 2050^1^. A remarkable increment in the incidence rate of breast cancer has been noted worldwide. Moreover, the younger age group now tends to be affected by breast cancer. The effective method for management of breast cancer will be controlling morbidity along with incidence rate.

According to WHO classification, breast cancer has several histological subtypes; however, due to different molecular and clinicopathological features, triple-negative breast cancer (TNBC) is categorized as high-grade invasive cancer within “special types”^2^. The TNBC lacks estrogen and progesterone receptor on the cell surface and lacks a protein called HER2. TNBC alone accounts for 10–15% of total cases, with 25% of breast cancer-related morbidity^3–5^. The diagnosis of TNBC is based on invasive methods, including morphological imaging followed by immunohistochemistry (IHC). However, detection of TNBC by the IHC method is costly and invasive and could produce false positive reports. For the reduction of false positive reports, fluorescent in situ hybridization (FISH) and HER2 gene amplification are also recommended by clinicians making it more costly to the diagnosis of patients^6,7^.

Moreover, the functional relevance of several genes was identified to understand the molecular features, diagnostic, prognostic, and therapeutic potential in the management of TNBC. However, none of them showed potential for being used in the diagnosis and management of TNBC. Therefore, there is an urgent need for minimally invasive biomarkers that have diagnostic and prognostic potential in the management of TNBC cases.

The miRNA are small non-coding RNA of 22–24 nucleotides in size that are aberrantly expressed in several carcinomas. Several mechanisms are involved in the dysregulated expression of miRNA, including epigenetic regulation, deletion/amplification, and failure of the miRNA biogenesis process^8^. Upregulated miRNAs are termed oncogenic, while downregulated miRNAs are tumor suppressors in nature. Moreover, aberrant expression of these miRNAs is associated with tumor progression by regulating biologically important pathways such as ABC transporter. apoptosis, cell cycle, and signalling pathways^9^.

Our candidate miRNA has been previously studied in breast cancer with respect to role of these miRNA in progression of EMT and metastases, however, there diagnostic potential specific to TNBC has not been examined yet^10,11^. Shichao et al. reported upregulation of miRNA-21 in breast cancer and have 89.0% AUC with 79.0% sensitivity and 66.0% specificity^12^. Moreover, Xinquan et al. reported the dysregulated expression of miRNA-21 can predict survival of TNBC patients^13^. In another study Fang et al. revealed miRNA-21 promotes cell proliferation and invasion in TNBC through inhibition of PTEN^14^. Similarly, miRNA-155 was elevated in breast cancer with an AUC of 91.0% with 87.0% sensitivity and 82.0% specificity^15^.miRNA-based regulatory role in breast cancer has been widely studied. Previously, the oncogenic role of miRNA-155 was well illustrated in most cancer, whereas, miRNA-155 regulates apoptosis, proliferation, and EMT and can regulate carcinogenesis. Similarly, other study reported the downregulated expression of miRNA-205 in triple negative breast cancer as compared to luminal A/B, HER2 + subtype of breast cancer^16–19^.

In the present study, we tried to evaluate the miRNA association with breast cancer by enrichment of miRNA-Target genes and functional enrichment analysis of miRNA-Target genes for TNBC cases. Moreover, significantly enriched miRNA was further selected for expression analysis in TNBC cases and healthy patients sample using qRT-PCR. The diagnostic potential of miRNA was further analysed using the receiver operating characteristic curve (ROC curve) based on expression data of miRNA. Moreover, the prognostic potential of miRNA was further evaluated by correlating miRNA expression data with different clinicopathological features.

## Methodology

### Sample recruitment, clinicopathological characteristics and study design

Prior to sample collection the ethical approval was obtained from the Institute Ethical Committee( Ref. no: NC/BIR/ORC/2020/610) of Neurosurgery Clinic, Birgunj, Nepal. The patients immunohistochemistry (IHC) of ER, PR, and HER2 data was obtained from the source hospital and based on ER, PR, and HER2 immunohistochemistry results 139 TNBC subjects were selected. We excluded participants who have undergone the hormone therapy, targeted treatments and chemotherapy process. The patients demographic and clinicopathological data were further obtained from source hospital (Table 1). All TNBC cases and healthy control samples was recruited during the May-2020 to June-2022 from Nepal and Bihar, India attending OPD of Department of Oncology, Neurosurgery Clinic, Birgunj, Nepal with dually signed patients informed consent form. The sample of TNBC and healthy patient’s preoperative blood samples were collected, and serum-based miRNA was isolated simultaneously upon collection of blood samples and stored at − 80 °C for further analysis. The study was performed according to Principle of the Declaration of Helsinki.

**Table 1:** Represent clinical characteristics of patients enrolled in this study.

Variables	Case (n = 139)	Control (n = 51)	Relative expression of miR-205 (2ΔΔCT ) ± SD	Relative expression of miR-155 (2-ΔΔCT ) ± SD	Relative expression of miR-21 2-ΔΔCT ± SD
Age, n (%)					
< 45	54 (38.8)	26 (53.3)	− 4.64 ± 3.08	5.90 ± 2.82	5.32 ± 3.27
≥ 45	85 (61.1)	27 (46.6)	− 5.40 ± 4.36	7.15 ± 3.12	5.24 ± 3.54
P-Value			ns	ns	ns
Distant metastasis, n (%)					
Absent	65 (46.7)	–	− 4.52 ± 3.20	5.10 ± 4.98	5.07 ± 2.23
Present	74 (53.2)	–	− 7.80 ± 6.25	8.40 ± 4.91	8.37 ± 3.21
P-Value			< 0.0001*	< 0.0001*	< 0.0001*
TNM stage, n (%)					
I–II	78 (56.1)	–	− 5.30 ± 3.30	5.21 ± 3.55	4.64 ± 3.87
III–IV	61 (43.8)	–	− 9.24 ± 5.10	9.89 ± 4.77	7.93 ± 6.72
P-Value			< 0.0001*	< 0.0001*	< 0.0001*
Menopause, n (%)					
Yes	85 (61.2)		− 4.50 ± 4.26	7.51 ± 4.68	6.33 ± 4.43
No	54 (38.8)		− 3.48 ± 2.26	4.31 ± 2.78	5.17 ± 3.20
P-Value			ns	ns	Ns
Serum CA-15–3 (U/ml)	184 ± 329.1	33.8 ± 61.4			

Statistical significance was obtained using Mann Whitney U-test. Data represent mean ± standard error on the mean (SEM). *represent statistical significance.

**Figure 1:**
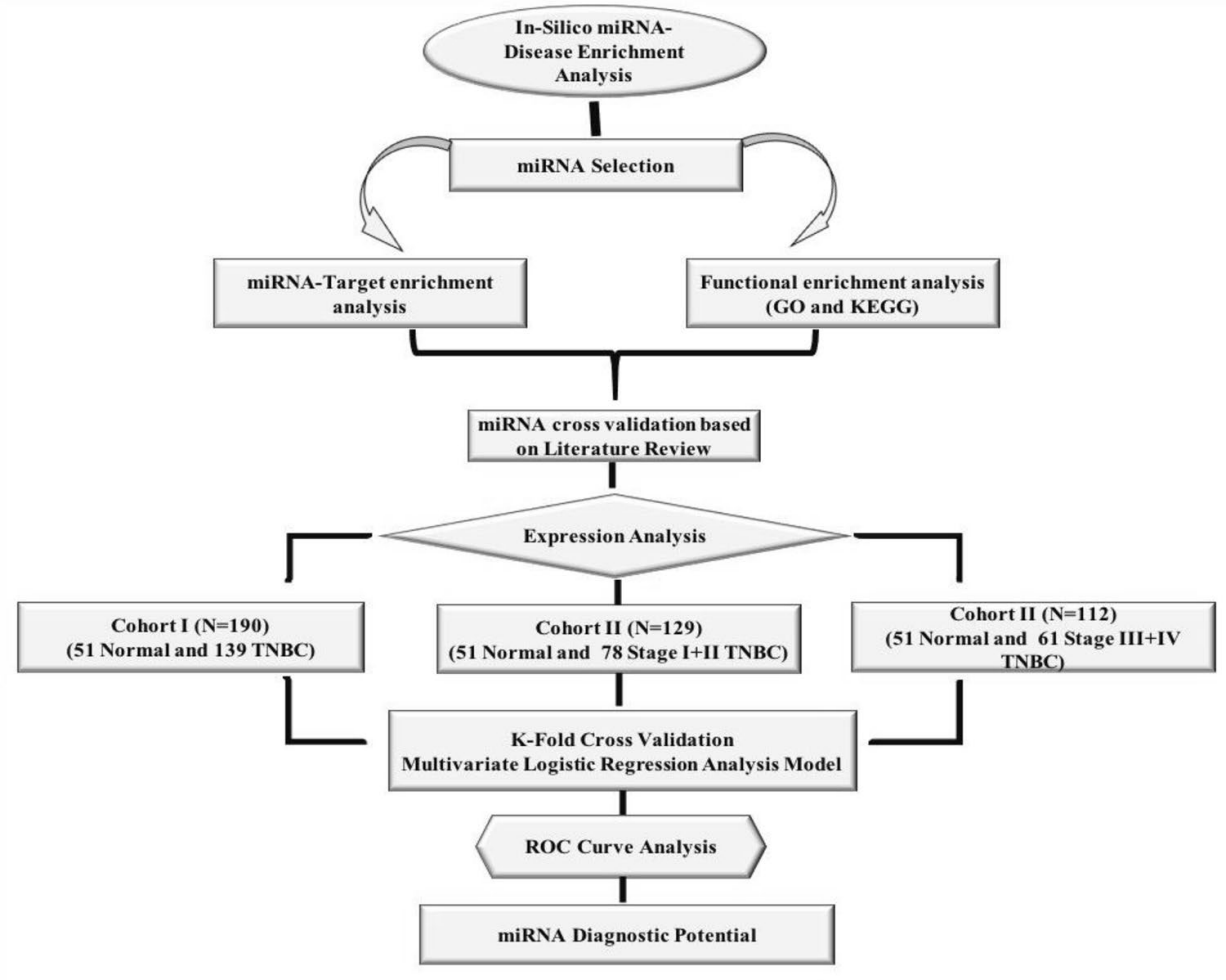
Diagrammatic representation showing present experimental study.

During this study our aim was to identify the differential expression of candidate miRNAs and there diagnostic potential in mixed stage as well as early and late stage TNBC patient’s group compared to control samples. Moreover, we were also interested to evaluate how does the change in expression of selected miRNA in different clinicopathological features (including the menopausal status, distant metastasis and age group) contribute towards disease progression. Therefore, we further, bifurcated our 139 TNBC samples into three cohorts {Cohort 1 (mixed cases; n = 139 and n = 51 control samples), Cohort 2 (78 TNBC (n = 35 stage-I and n = 43 stage-II), and cohort III (n = 31 stage-III and n = 30 stage-IV)}. The overall study design is represented in Fig. 1. During analysis the control samples (n = 51) was constant in each cohort.

### miRNA selection criteria

In-silico analysis was conducted for identification of miRNA involve in TNBC progression using freely available online databases (miRDB. TargetScanHuman7.2, miRnet 2.0) and further we also performed extensive literature survey for the selection of our candidate miRNA. These databases have extensive data mining capability with respect to our queries. Therefore, we first evaluated the miRNA associated with breast cancer and most common miRNA from all tools were selected manually. Further, we narrowed our queries to the TNBC subtype of breast cancer and built a miRNA-disease enrichment network. Next, we narrow down our search to see the interaction of miRNA-Target genes and performed GO and KEGG analysis to reveal their role in disease progression. To assure the right selection miRNA, we correlated in-silico analysis results with recently published article describing the role and dysregulated expression of our candidate miRNA in TNBC subtype of breast cancer.

### miRNA isolation from patient’s serum sample

Total miRNA was extracted from 139 TNBC cases and 51 healthy patient’s serum samples using MagMAX™ mirVana™ Total RNA Kit (cat no: A27828, ThermoFisher) according to the manufacturer's instruction. Moreover, 3.5 µl of Spike-In Control (1.6 × 10^8^ copies) (cel-miR-39) was mixed with samples for normalization during qRT-PCR. Total isolated miRNA was further measured using a nanodrop spectrophotometer (Thermofisher, USA). The quantity of isolated miRNA in each serum sample within a range of 0.6–2.8 µg.

### miRNA expression analysis

Further, miRNA relative expression in the patient’s serum sample was evaluated using RT-qPCR. cDNA was prepared using miScript® II RT Kit (cat no: 218160, Qiagen) according to the manufacturer’s protocol. A total of 0.8 µg of miRNA from each sample was converted to cDNA and kept in − 20 °C till further analysis. The expression level of candidate microRNA was evaluated using SYBR® Green PCR Kit (cat no: 218073, Qiagen) with a specific primer assay (miScript Primer Assay, cat no: 218300, Qiagen) by RT-qPCR machine (MiniOptician, BioRad). For successive RT-qPCR reactions, 10 ng/µl of cDNA was used. Each sample was quantified in triplicate following the manufacture's protocol. The cycling condition for RT-qPCR reaction was initial activation at 95 °C for 15 min, following cycling conditions: denaturation (94 °C for 15 s), annealing (55 °C for the 30 s), and extension (70 °C for 30 s, for 40 cycles). miRNA-191 and cel-miR-39 was used as internal control for normalization following ΔCt (CtmiRNA-0.5*(Ctcel-miR-39 + CtmiR-191)^20^.

### Statistical analysis

Further, the quantitative fold change of each miRNA was calculated by Livak and Schmittgen method (2-ΔΔCq)^21^. All expression values were denoted as mean and standard deviation, while categorical data were presented as percentages and count. Differences between continuous variables were evaluated by the Mann–Whitney U test. Spearman’s rank-order correlation was used to investigate the correlation between clinicopathological features and an expression of miRNA. The Stratified k-fold cross-validation framework multivariate binary logistic regression was used to build a miRNA-disease association model facilitating the assessment of the predicted probability of miRNA and miRNA-panel, which was employed to construct the ROC curve analysis revealing sensitivity and specificity using RStudio 2023.09.0. SPSS® (Version-27, SPSS Inc., USA) was used to evaluate the statistical significance of each test, and GraphPad Prism (Version 9.0) was used to draw graphs. All statistical analyses were two-sided, and the significance of the analysis was considered if P-value < 0.05.

### Ethics approval and consent to participate

Study was approved by Institute ethical committee of Neurosurgery Clinic, Birgunj, Nepal (NC/BIR/ORC/2020/610), and informed consent form was dually signed by patients.

## Results

### Pathological features of TNBC patients.

Total 190 patients serum samples (TNBC = 139 and Healthy = 51) were used in the present study. The age of patient’s ranges between 39 to 60 years. The 139 TNBC cases includes 35 stage-I (25.1%), 43 stage-II (30.9%), 31 stage-III (22.3%), and 30 stage-IV (21.5%) samples. Further we built three different cohorts based on 139 TNBC samples stages and their clinicopathological features. Cohort-I consists of all TNBC (n = 139) and control (n = 51) samples irrespective of stages, cohort II consist of stage I + II TNBC cases (n = 78; 56.11%), while cohort III consist of stage III + IV TNBC cases (n = 61; 43.8%). Moreover, Samples were bifurcated by metastatic nature (74 metastatic (53.2%) and 65 non-metastatic (46.7%) and menopausal status (91 patients had menopause (65.4%), while 48 patients were in a premenopausal state (34.5%)). The overall patient clinical features are elaborated in Table 1.

### Selection of miRNA

**Figure 2:**
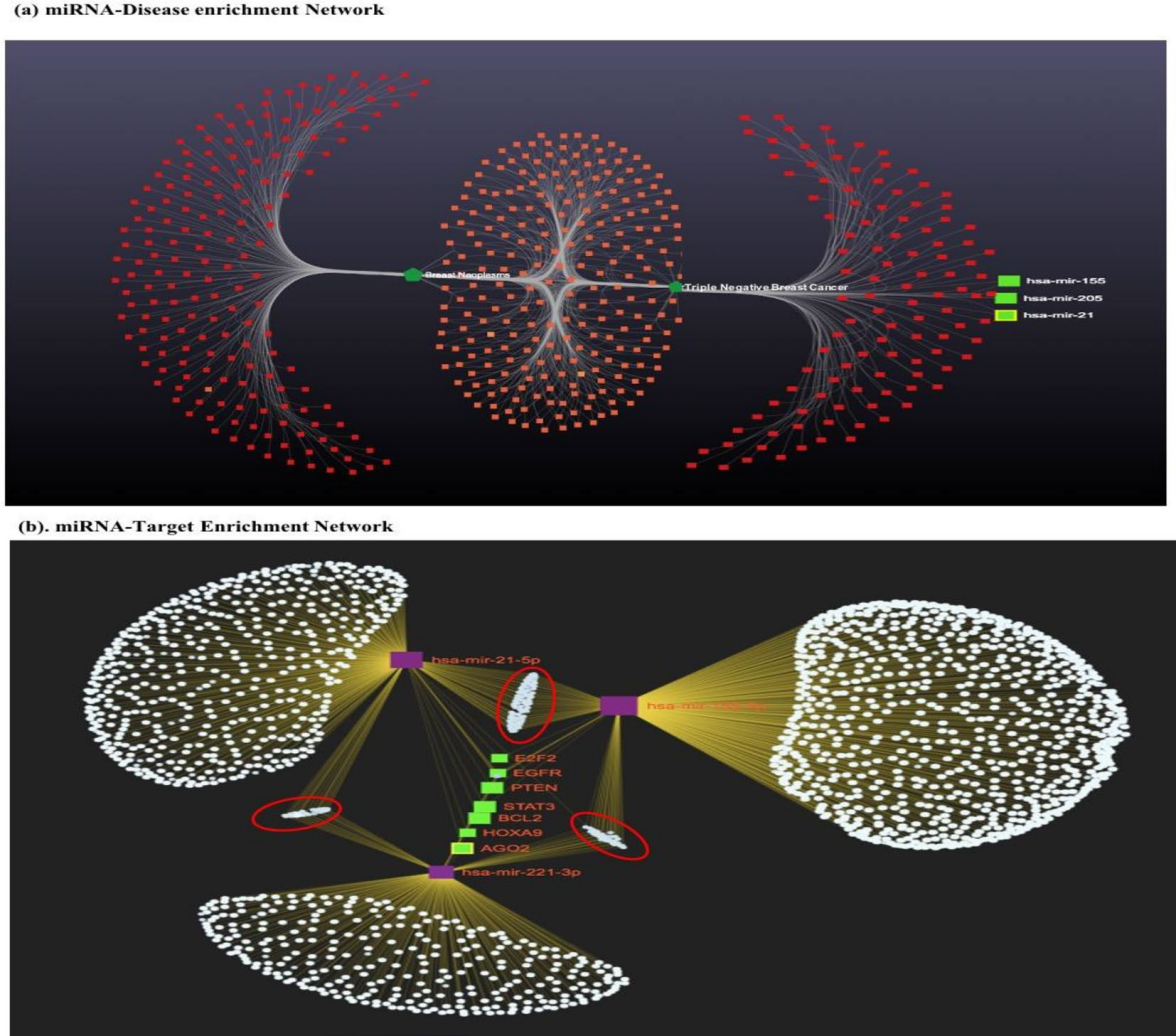
Represents bioinformatics based miRNA-disease enrichment, Target enrichment and function annotation of selected miRNA-Target genes in breast cancer (**a**) miRNA-disease enrichment analysis revealed miRNA-205, miRNA-155 and miRNA-21 was significantly enriched in breast cancer (based on betweenness, enrichment score and padj < 0.05) and (**b**) Highly enriched candidate miRNA-target genes are presented in green colour (padj < 0.001) and other significantly enriched genes are marked in red circle (padj < 0.05). The disease network analysis and miRNA-Target enrichment analysis was performed using miRNet 2.0 online tools (www.mirnet.ca), and the statistical analysis was performed using R programming based package of miRNet 2.0 available on GitHub.

We performed In-Silico analysis for the identification of miRNA exclusively associated with breast cancer study. Therefore, miRNA association with the disease was evaluated using three independent online databases (miRDB. TargetScanHuman7.2, miRnet 2.0). As a result, 510 miRNA were significantly enriched in breast cancer (padj-value < 0.05) (Fig. 2a.). The significantly enriched overlapping common miRNA from each database were sorted. Further, we set queries “only to TNBC subtype” and common miRNA (n = 210; padj < 0.05) were used to build the miRNA-disease enrichment network analysis with default parameter using miRNet 2.0 a freely available online tools (www.mirnet.ca)^22^. Based on betweenness, enrichment score, and padj-value (padj < 0.05) we selected three candidate miRNA (miRNA-205, miRNA-155, and miRNA-21) for further analysis. Before moving ahead we also reviewed the recently published paper confirming the role of dysregulated miR-205, miR-155 and miR-21 in TNBC progression. Selected candidate miRNAs were evaluated for target enrichment analysis using miRnet 2.0 tools. The result of this analysis helps us to identify the putative target genes of miRNA exclusively associated with breast cancer disease progression. More than 7000 genes were enriched in our analysis, and upon applying the filter (exclusively in breast cancer and Padj < 0.05), only 250 genes showed to have significant enrichment scores with TNBC (Fig. 2b), and genes such as E2F2, EGFR, PTEN, STAT3, BCL2, HOXA9, and AGO2 shown to have higher log2FC with a padj-value and betweenness score in the enriched network (Fig. 2b).

### Functional annotation of selected miRNA in breast cancer progression

After the selection of miRNA and Targets enrichment, we performed functional annotation of miRNA-Target genes (KEGG and Gene Ontology) using the DAVID database(https://david.ncifcrf.gov/)^23^. Functional annotation could reveal the association of miRNA-Target genes in breast cancer progression. KEGG Pathway enrichment analysis revealed eight pathways ‘Pathways in cancer’, ‘PI3K-Akt signalling pathway’, ‘miRNA in cancer’, ‘MAPK signalling pathway’, ‘Hepatitis B’, ‘Salmonella infection’, and ‘Breast cancer’ were highly enriched terms (Fig. S1). Similarly, Gene Ontology (GO) analysis revealed top ten terms in each Biological process (BP), Cellular Component (CC) and Molecular Function (MF). Most importantly, cytosol (GO:0,005,829), regulation of transcription from RNA polymerase II promoter (GO:0,006,357), negative regulation of gene expression (GO:0,010,629), metal ion binding (GO:0,046,872), and RNA binding (GO:0,003,723) were highly enriched terms in MF, CC and BP of Gene Ontology (Fig. S2).

### Relative expression analysis of candidate miRNA

**Figure 3:**
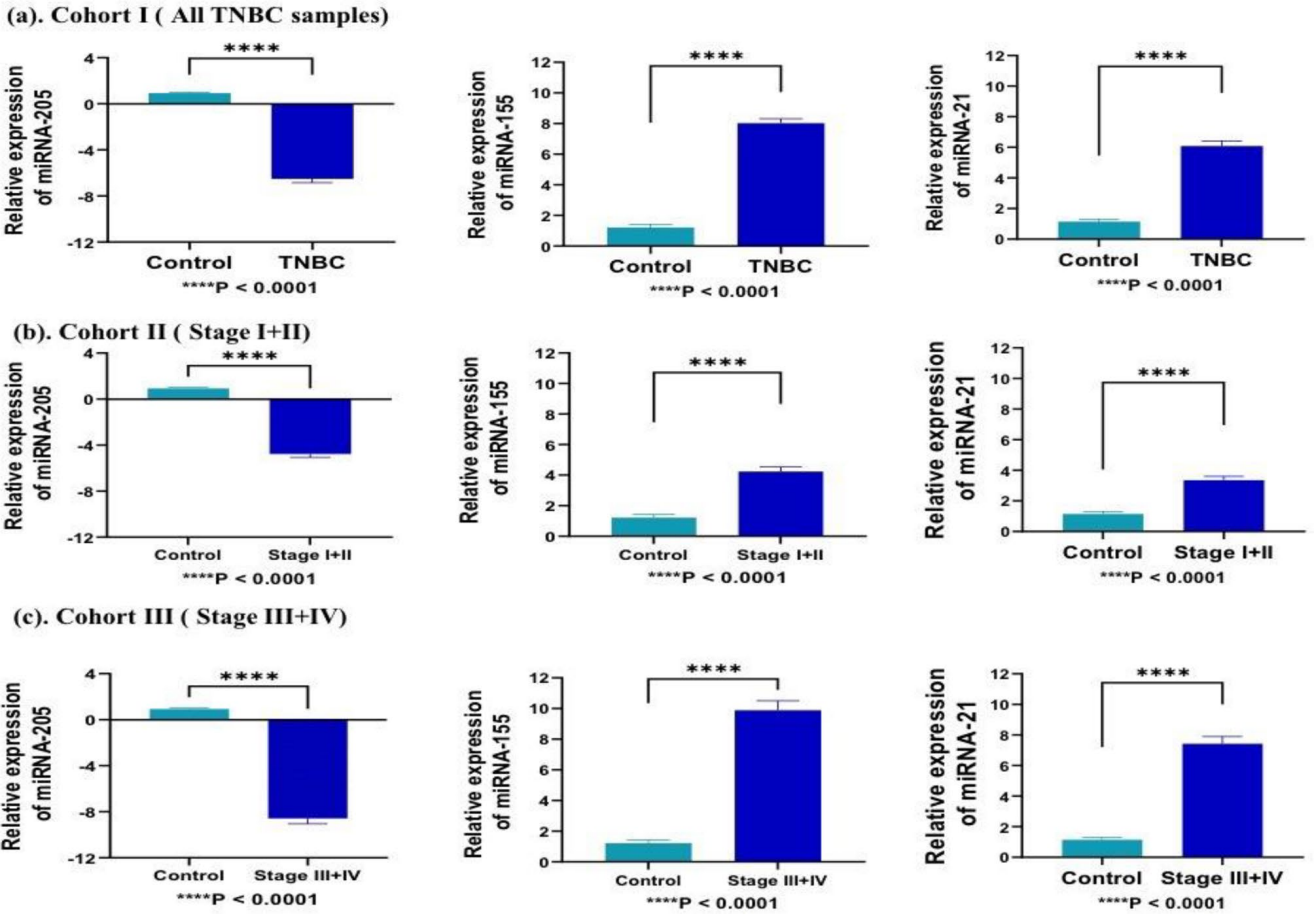
Represents relative expression of selected miRNA in different cohorts compared to healthy patients samples. (**a**) In cohort-I (TNBC = 139, control = 51), miR-155 and miRNA-21 significantly overexpressed (7.26; *P* < 0.0001, and 6.05; *P* < 0.0001)., while miRNA-205 was downregulated (− 7.03; *P* < 0.0001) in all TNBC samples as compared to control (**b**) In cohort-II (TNBC = 78, control = 51), miRNA-205 was downregulated, while, miR-155 and miRNA-21 significantly overexpressed in early stage (stage I + II) samples as compared to control with fold change of − 5.30 (*P* < 0.0001), 5.21 (*P* < 0.0001), and 4.68 (*P* < 0.0001), respectively (**c**) In cohort-III(TNBC = 61, control = 51), miRNA-205 was downregulated, while, miR-155 and miRNA-21 significantly overexpressed in late stage (stage III + IV) samples as compared to control with fold change of − 9.24 (*P* < 0.0001), 9.89 (*P* < 0.0001), and 7.93 (*P* < 0.0001), respectively. Statistical significance was determined by *p* < 0.05 by Mann Whitney U-test. Data represented as mean and standard deviation, ****P < 0.0001.

The quantitative expression analysis of miRNA (miRNA-205, miRNA-155, and miRNA-21) was performed by RT-qPCR in TNBC and in contrast to control samples. In cohort-I (TNBC = 139, control = 51), the expression level of miRNA-155 and miRNA-21 was overexpressed, and miRNA-205 was downregulated in TNBC cases with respect to controls samples with a fold change of 7.26 (P < 0.0001), 6.05 (*P* < 0.0001), − 7.03 (*P* < 0.0001) respectively (Fig. 3a). In cohort-II (Stage I + II; TNBC = 78, control = 51), the expression of miRNA-155 and miRNA-21 was overexpressed, and miRNA-205 was downregulated in TNBC cases with respect to controls with a fold change of 5.21 (*P* < 0.0001), 4.68 (*P* < 0.0001), and − 5.30 (*P* < 0.0001), respectively (Fig. 3b). Similarly, In cohort-III (stage III + IV; TNBC = 61, control = 51), the expression of miRNA-155 and miRNA-21 was significantly overexpressed, and miRNA-205 was downregulated in TNBC cases with respect to controls with a fold change of 9.89 (*P* < 0.0001), 7.93 (*P* < 0.0001), − 9.24 (*P* < 0.0001), respectively (Fig. 3c).

### Evaluation of diagnostic potential of candidate miRNA

Further, to predict the diagnostic potential of miRNA, built separate model for each of the cohorts using R-software. The model was based on Stratified *K-*Fold Cross-Validation framework implying the multivariate binary logistic regression analysis. For each cohort, we built separate model where randomly 75% of data were selected for training dataset while remaining 25% of data was used for validation in test dataset. Each trained model of respective cohort (cohort-I, cohort-II and cohort-II) was exposed to test dataset and performance of model was evaluated. The performance of model from cohort-I to predict TNBC was found to have 73.47% accuracy, 0.445 kappa value with statistical significance (*p* < 0.05). Similarly, the cohort-II have accuracy of 84.22% with 0.711 kappa value while cohort-III showed to have 69.32% accuracy with 0.324 kappa value (Table 2). Since, our model for each cohort doesn’t showed any overfitting issue we further moved to analyse the association of each miRNA in disease progression, for this the multivariate regression analysis of each model was evaluated. In our k-fold regression model, miRNA-205, miRNA-155, and miRNA-21 were coupled with disease outcome in each of the tested cohorts with *p* < 0.05. During analysis, the 95% CI was used as the accuracy of regression coefficients, while statistical significance was denoted as a p-value (*P* < 0.05) (Table 3).

**Table 2:** Represent the accuracy of model from each cohort in predicting the disease state using the k-fold based cross-validation framework with multivariate binary logistic regression analysis.

Model	Accuracy (%)	K	95% CI	Sen (%)	Spe (%)	P value
Cohort-I	73.47	0.445	0.611–0.854	82.1	66.9	0.0001
Cohort-II	84.22	0.711	0.341–0.988	86.8	78.8	0.00001
Cohort-III	69.32	.324	0.548–0.862	81.7	66.77	0.001

The kappa value ranges between 0 to 1, higher the k value better the model in predicting the disease. *K* Kappa value, *Sen* sensitivity, *Spe* specificity *P value* probability value.

**Table 3:** Represents binary logistic regression analysis of miRNA expression in three different cohorts. In all three cohorts miRNA disease association model have significant p-value (*p* < 0.05) determining the miRNA involvement in disease progression.

miRNA Types	(a) Cohort-I (139 TNBC, 51 Control)			(b) Cohort-II (78 TNBC, 51 Control)			(c) Cohort-III (61 TNBC, 51 Control)		
	Regression Coefficient (B)	95% CI	P-Value	Regression Coefficient (B)	95% CI	P-Value	Regression Coefficient (B)	95% CI	P-Value
miR-205	1.076	1.36–6.30	0.001	1.09	1.34–6.64	0.007	0.828	1.25–4.18	0.026
miR-155	0.501	1.27–2.14	0.001	0.444	1.13–2.14	0.006	0.487	1.18–2.23	0.027
miR-21	1.152	1.71–5.85	0.001	0.792	1.23–2.09	0.007	0.789	1.34–3.60	0.029

*B* regression coefficient, *CI* confidence Interval, *represents highly significant difference.

**Figure 4:**
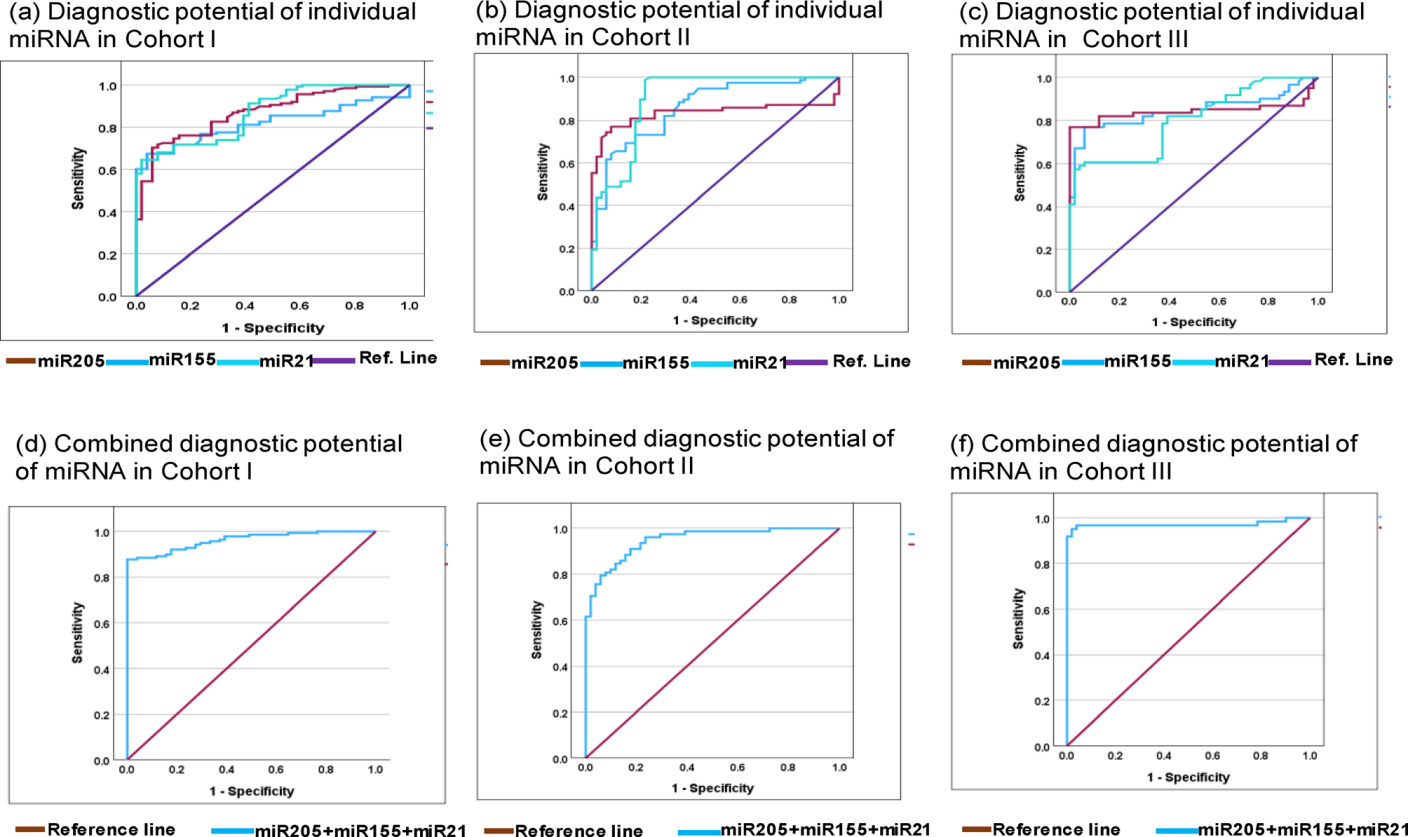
Represents ROC curve analysis of individual miRNA and miRNA-panel toward diagnosis of Triple negative breast cancer. (**a**) Represent diagnostic potential of individual miRNA-205, miRNA-155 and miRNA-21 in cohort-I with AUC at 95% CI was 81.9% (0.761–0.876 ), 87.0% (0.819–0.921 ), and 86.9% (0.919–0.920) respectively. (**b**) Represent diagnostic potential of individual miRNA-205, miRNA-155 and miRNA-21 in cohort-II with AUC at 95% CI was 83.3% (0.756–0.909), 85.8% (0.794–0.923), and 89.9% (0.839–0.960) respectively. (**c**) Represent diagnostic potential of individual miRNA-205, miRNA-155 and miRNA-21 in cohort-II with AUC at 95% CI was 84.3% (0.758–0.928), 84.7% (0.770–0.923), and 80.0% (0.720–0.880) respectively. (**d**) Represents combined diagnostic potential of miRNA-205 + miRNA-155 + miRNA-21 in cohort-I with AUC at 95% CI was 96.1% (0.938–0.985). (**e**) Represents combined diagnostic potential of candidate miRNA with AUC at 95% CI was 94.9% (0.915–0.983). (**f**) In cohort-III, the combined diagnostic potential candidate miRNA with AUC at 95% CI was 97.1% (0.933–1.009). *AUC* area under curve, *SEN* sensitivity, *SPE* specificity.

Next, we evaluated the diagnostics capability of each individual miRNA from each cohort using ROC curve analysis. The diagnostic capability of miRNA was measured as AUC, sensitivity, and specificity with an optimal cut-off value at 95% CI. In cohort-I, the AUC, sensitivity, specificity of individual miRNA-205, miRNA-155, and miRNA-21 was 81.9% (sensitivity = 77.5%, specificity = 66.4%), 87.0% (sensitivity = 87.7%, specificity = 63.7%), and 86.9% (sensitivity = 79.0%, specificity = 64.5%) (Fig. 4a). Moreover, in cohort-II the AUC, sensitivity, specificity of individual miRNA-205, miRNA-155, and miRNA-21 was 83.3% (sensitivity = 84.6%, specificity = 82.5%), 85.8% (sensitivity = 84.1%, specificity = 81.3%), 89.9% (sensitivity = 88.5%, specificity = 80.4%) (Fig. 4b). While, in cohort-III the AUC, sensitivity, specificity of individual miRNA-205, miRNA-155, and miRNA-21 was 84.3% (sensitivity = 86.9%, specificity = 82.4%), 84.7% (sensitivity = 90.2%, specificity = 80.4%), and 80.0% (sensitivity = 95.1%, specificity = 72.5%) (Fig. 4c).

In addition, we evaluated the combined diagnostic capabilities of miRNA-205, miRNA-155, and miRNA-21 from each cohort. Therefore, predicted probability of each model was used to evaluate the diagnostic potential of miRNA-panel in predication of TNBC using ROC-AUC analysis. The combined diagnostic value of miRNA-205, miRNA-155, and miRNA-21 in cohort-I was AUC = 96.1% (sensitivity = 89.9%, specificity = 85.6%) (Fig. 4d). Similarly, in cohort-II, the combined AUC was 94.9% (sensitivity = 91.0%, specificity = 82.3%) (Fig. 4e), while, in cohort-III, the AUC was 97.1% with 96.7% of sensitivity and 81.2% of specificity (Fig. 4f). The detailed diagnostic potential of individual and combined miRNA panel is elaborated in Table 4.

**Table 4:** Represents ROC curve analysis of miRNAs in three different cohorts.

miRNA Types	(a) Cohort-I					(b) Cohort-II					(c) Cohort-III				
	AUC (%)	SEN (%)	SPE (%)	95% CI	CV	AUC (%)	SEN (%)	SPE (%)	95% CI	CV	AUC (%)	SEN (%)	SPE (%)	95% CI	CV
miR-205	81.9	77.5	66.4	0.761–0.876	1.270	83.3	84.6	82.5	0.756–0.909	0.830	84.3	86.9	82.4	0.758–0.928	0.515
miR-155	87.0	87.7	63.7	0.819–0.921	1.245	85.8	84.1	81.3	0.794–0.923	0.880	84.7	90.2	80.4	0.770–0.923	0.410
miR-21	86.9	79.0	64.5	0.919–0.920	1.121	89.9	88.5	80.4	0.839–0.960	0.254	80.0	95.1	72.5	0.720–0.880	0.765
Combined	96.1	89.9	85.6	0.938–0.985	0.412	94.9	91.0	82.3	0.915–0.983	0.321	97.1	96.7	81.2	0.933–1.009	0.101

*AUC* area under curve, *SEN* sensitivity, *SPE* specificity, *CV* optimal cut-off value, *CI* confidence interval.

### Comparative analysis of miRNA expression with different pathological features in TNBC cases

**Figure 5:**
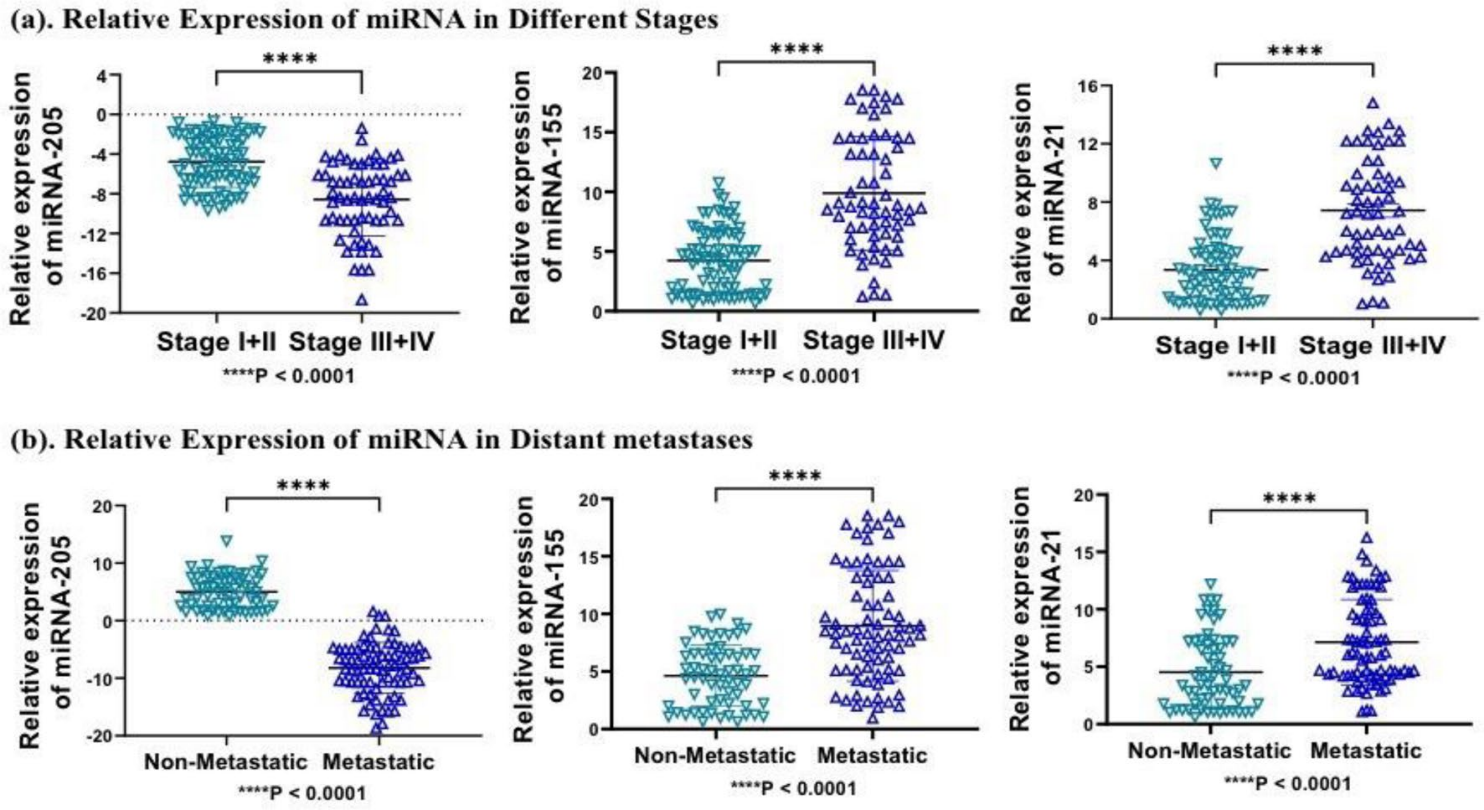
Represents relative expression of miRNA in different stages and distant metastatic nature of samples (**a**) miRNA-205, miRNA-155 and miRNA-21 was significantly overexpressed in combined stage III + IV compared to stage I + II (*P* < 0.0001). (**b**) Similarly, miRNA-205, miRNA-155 and miRNA-21 was significantly overexpressed in metastatic samples compared to non-metastatic samples (*P* < 0.0001). Statistical significance was determined by *p* < 0.05 by Mann Whitney U-test. Data represented as mean and standard deviation, ****P < 0.0001.

Further, we evaluated the expression pattern of candidate miRNA in different clinicopathological features, including stages, metastases, and menopausal status of patients. Relative expression of miRNA-155 and miRNA-21 was significantly upregulated, while miRNA-205 was downregulated in combined stage III + IV, with fold changes of 9.89, 7.99, and − 9.24 compared to combined stage I + II with a fold change of 5.2, 4.64 and − 5.30, respectively with significant P value (*P* < 0.001) and can discriminate between late stage patients of TNBC with early-stage patients (Fig. 5a). Similarly, metastatic samples show higher expression of miRNA-155, and miRNA-21 compared to non-metastatic samples with greater fold change and significant P-value (*P* < 0.0001), while miRNA-205, was negatively expressed in metastatic samples (*P* < 0.0001) (Fig. 5b). In addition, miRNA-205, miRNA-155, and miRNA-21 expression were not significant in the menopausal status of patients compared to the non-menopausal status of patients.

Further, samples were bifurcated according to patient's clinicopathological features such as age, distance metastasis, and menopausal status and we evaluated the dysregulated expression of the each miRNA in individual parameter. This analysis would tell us how does miRNA behave in each pathological condition. Further, we applied Spearman’s rank-order correlation analysis to evaluate the correlation between change in expression of miRNA with each pathological features. In cohort-I, expression level of miRNA-205, miRNA-155, and miRNA-21 showed to have significant negative correlation with the age (r = − 0.224, r = − 0.382, r = − 0.386), while positive correlation with menopausal status (r = − 0.408, r = 0.288, and r = 0.645), and distance metastasis (r = 0.312, r = 0.309, and r = 0.532). In cohort-II, the expression of miRNA-205, miRNA-155, and miRNA-21 showed negative correlation with age (r = − 0.564, r = − 0.280, r = − 0.226), while positive correlation was seen with menopausal status (r = 0.221, r = 0.178, and r = 0.243) and distance metastases (r = 0.243, r = 0.321, and r = 0.379. Similarly, in cohort-III, all candidate miRNA (miR-205, miR-155 and miR-21) showed a strong negative correlation with age (r = − 0.398, r = − 0.266, and r = − 0.321), while miRNA-205, miRNA-155, and miRNA-21 showed a strong positive correlation with menopausal status (r = 0.462, r = 0.388, and r = 0.427) and distant metastases (r = 0.639, r = 0.765, and r = 0.785) respectively (Table 5).

**Table 5:** Correlation analysis of miRNA-205, miR-155 and miRNA-21 expression with clinicopathological features.

Parameters	(a) cohort-I (139 TNBC, 51 Control)				(b) Cohort-II (78 TNBC, 51 Control)				(c) Cohort-III (61 TNBC, 51 Control)			
		miR-205	miR-155	miR-21		miR-205	miR-155	miR-21		miR-205	miR-155	miR-21
Age	r p	− 0.224 0.05*	− 0.382 0.01*	− 0.386 0.01*	r p	− 0.564 0.001*	− 0.280 0.05*	− 0.226 0.05*	r p	− 0.398 0.01*	− 0.266 0.05*	− 0.321 0.05*
Menopausal status	r p	0.408 0.001*	0.288 0.05*	0.645 0.0001*	r p	0.221 0.05*	0.178 0.05*	0.243 0.05*	r p	0.462 0.001*	0.388 0.01*	0.427 0.001*
Distant metastases	r p	0.312 0.001*	0.309 0.01*	0.532 0.0001*	r p	0.243 0.05*	0.321 0.01*	0.379 0.01*	r p	0.639 0.0001*	0.765 0.0001*	0.785 0.0001*

(a) In cohort-I, miRNA-205, miRNA-155 and miRNA-21 were negatively correlated with age, while, all miRNA was positively correlated with menopausal status and distance metastases with significant P-value (*p* < 0.05) (b) Similarly in cohort-II, miRNA expression showing negative correlation with age and positive correlation with the menopausal status and distance metastases with significant P-value (*p* < 0.05) (c) In cohort-III, all three miRNA showed negative correlation with age while, positive correlation with other two clinicopathological features with higher statistical significance. The Non-parametric Spearman’s Rank correlation analysis was used to evaluate the correlation miRNA expression with different clinicopathological features.

r = regression coefficient, P = probability, *represents highly significant difference.

## Discussion

In-silico evaluation of miRNA shows the exclusive association of selected miRNA in breast cancer patients. Moreover, miRNA-target enrichment analysis uncovered several important target genes, such as E2F2, EGFR, PTEN, STAT3, BCL2, and HOXA9. Further, KEEG analysis revealed the involvement miRNA-Target genes in several important biological pathways, including “pathways in cancer, MAPK signaling pathways, PI3K-Akt signaling pathways, ErbB signaling pathways, and breast cancer”. In addition, Gene Ontology analysis revealed the participation of miRNA-target genes in multiple biological processes (negative regulation of gene expression, transcriptional regulation from RNA polymerase), molecular function (DNA binding, RNA binding, ATP binding), and cellular component (cytoplasm, chromatin, nucleus, and cytosol). These results suggest the implication of miRNA-Target genes in the pathogenesis and progression of TNBC. In context to the above finding, Fang et al. revealed regulation of PTEN by miRNA-21 leads to the promotion of proliferation and invasion capacity of TNBC cells^14^. Similarly, miRNA-155-based regulation of the anti-apoptotic gene (BCL2) has been reported^24^.

After appraising the role of candidate miRNA in disease progression further, we evaluated the expression of candidate miRNA in TNBC cases. Recruited samples were divided into three different cohorts based on stages. In cohort (mixed stage), cohort-II (stage I-II), and cohort-III (stage III-IV), the expression of miRNA-155 and miRNA-21 were significantly elevated in TNBC compared to healthy patients sample, while miRNA-205 was significantly downregulated in TNBC patients of all three cohorts compared to healthy patients sample (*P* < 0.0001). According to our results, dysregulated expression of our candidate miRNA could be used as an independent biomarker for evaluating TNBC cases. In present study, elevated expression of miRNA-155 and miRNA-21 and low levels of miRNA-205 in TNBC were consistent with previous studies^10,25,26^. Wang et al. reported frequent downregulation of miRNA-205 in TNBC. Moreover, reduced expression of miRNA-205 involved in EMT and invasion in TNBC by reducing the HMGB1-RAGE signaling mechanism^24^. Similarly, Piasecka et al. revealed low level of miRNA-205 enhances the EMT and invasion in TNBC^27^. Kong et al. reported overexpression of miRNA-155 enhances tumor angiogenesis and has diagnostics and prognostics values for TNBC^10^. Study by, Pasculli et al. also revealed the significant elevation of miRNA-155 in breast cancer^28^. Further, overexpression of miRNA-21 in triple-negative breast cancer and their involvement in proliferation and invasion has been well established^13,14^. It has been shown that upregulated miRNA-21 results in invasion, migration, and colony formation; however, Inhibition of miRNA-21 with RNA nanoparticles results in reduced TNBC cell migration and colony formation^29^.

We also evaluated the diagnostic potential of single miRNA and miRNA panels in different cohorts of TNBC. Our findings in mixed-stage TNBC samples revealed higher AUC, sensitivity, and specificity of miRNA-205, miRNA-155, and miRNA-21. Similar analysis on early-stage (stage I-II) and advanced-stage TNBC samples (stage III-IV) showed higher AUC, sensitivity, and specificity of candidate miRNA in predicting TNBC cases. The most prominent diagnostic value was obtained in early-stage samples (cohort-II) where each miRNA showed higher AUC, sensitivity, and specificity in predicting TNBC. Moreover, the combined diagnostic value of all three miRNA has greater AUC, sensitivity, and specificity than individual miRNA for predicting TNBC. In a recent studies higher predictive and prognostic value of miRNA-21 in the diagnosis of TNBC has been reported^14,30^. Moreover, Shichao et al. reported upregulation of miRNA-21 in breast cancer and have 89.0% AUC with 79.0% sensitivity and 66.0% specificity^12^. Similarly, miRNA-155 was elevated in breast cancer with an AUC of 91.0% with 87.0% sensitivity and 82.0% specificity^15^.miRNA-based regulatory role in breast cancer has been widely studied. Previously, the oncogenic role of miRNA-155 was well illustrated in most cancer, whereas, miRNA-155 regulates apoptosis, proliferation, and EMT and can regulate carcinogenesis.

In present study, we evaluated the expression of miRNA with clinical characteristics of TNBC patients. Our expression data analysis revealed that miRNA-155 and miRNA-21 have significantly greater fold change in advanced stage samples (stage III-IV) in contrast to early-stage samples (stage I-II) of TNBC. In addition, miRNA-205 was significantly downregulated in advance stage samples as compared to early stage samples) of TNBC. Moreover, we also evaluated the differential expression of miRNA-205, miRNA-155, and miRNA-21 in confirmed metastatic samples and non-metastatic samples. miRNA-205 was significantly downregulated in metastatic samples, while, miRNA-155 and miRNA-21 were significantly higher in metastatic samples correlating the potential of these miRNAs in differentiating the metastatic nature of breast cancer patients. In addition, the correlation of miRNA expression data with clinicopathological characteristics (age, menopausal status, and distant metastases) revealed a positive correlation with age, menopausal status, and distant metastases, except, miRNA-205 was negatively correlated with metastatic samples in cohort-I. In cohort-III, only distant metastases were shown to have a strong positive correlation with miRNA-155 and miRNA-21 expression, while miRNA-205 was negatively correlated with the metastatic nature of patients. In cohort II, the correlation was not significant. Correlation results in establishing the concept of miRNA roles in promoting metastasis by regulating several EMT-based markers.

Overall, this study establishes a proof of concept that dysregulated expression of miRNA in liquid biopsy could be used as minimally invasive biomarker for the diagnosis of TNBC cases. In addition, to the best of our knowledge, this is the first report to implicate the collective diagnostic potential of miRNA-205, miRNA-155, and miRNA-21 for the diagnosis of TNBC with significantly higher AUC, sensitivity, and specificity. However, this study has certain limitation, the miRNA selection should be performed with the inhouse sample using miRNA-Seq based expression analysis. In addition, potential of these miRNAs biomarker should be further validated in a larger cohort of samples, including tissue samples and serum samples of TNBC from a larger demographic area. Further molecular level analysis should be conducted for the better correlation of candidate miRNA role in TNBC progression.

Nevertheless, liquid biopsy-based circulating biomarker-based diagnostic test has to conquer several impediments before their clinical administration as a biomarker. A positive result is absolutely dependent upon the universal protocol for miRNA isolation, normalization, and effective quantitative expression analysis technique at a low cost.

### Supplementary Information

Supplementary Information.

## Data Availability

The data presented in this study are available on request from the corresponding author.
