# Supplementary material for: Impact of three miRNA signature as potential diagnostic marker for triple negative breast cancer patients
Source: Sci Rep. 2023 Dec 8;13:21643. doi: 10.1038/s41598-023-48896-7 (PMC10703933; doi:10.1038/s41598-023-48896-7)
Supplement: Supplementary file 1 — Supplementary Information. [file 41598_2023_48896_MOESM1_ESM.pdf]

Supplementary Figure

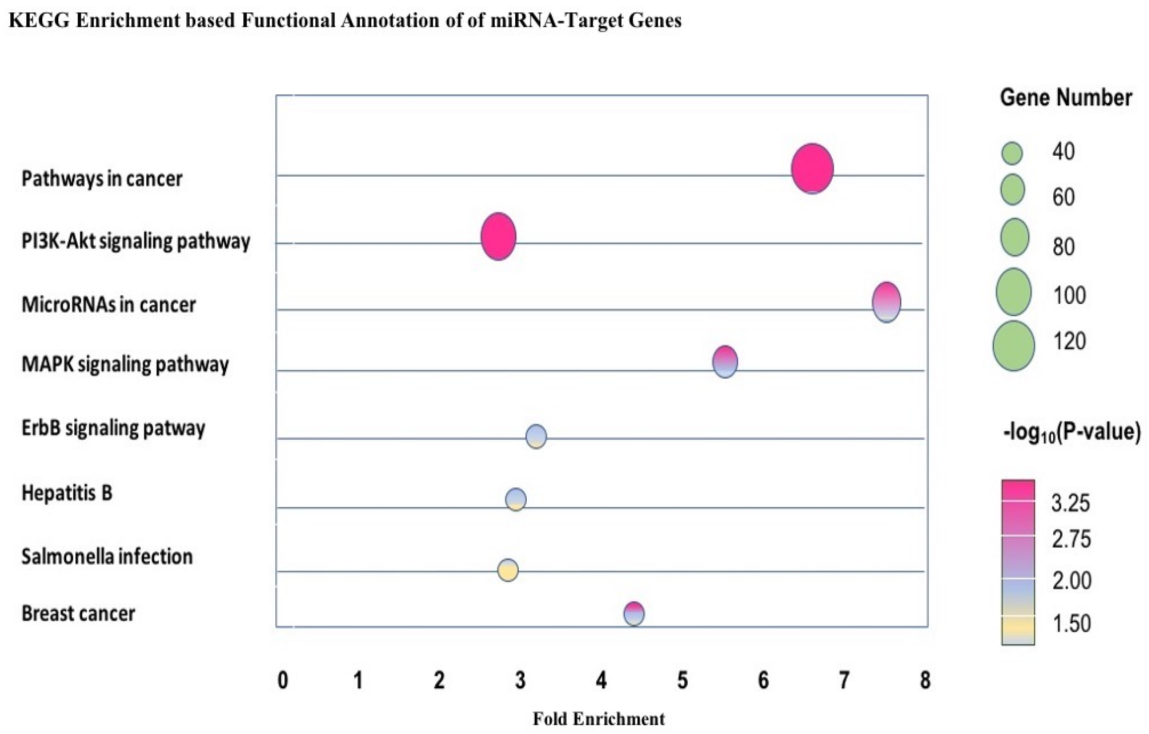

Figure S1. Represents functional enrichment analysis based on Kyoto Encyclopedia of Genes and Genomes analysis (KEEG) using DAVID online tools, revealing top eight highly enriched pathways

Gene Ontology analysis of candidate miRNA-Target Genes

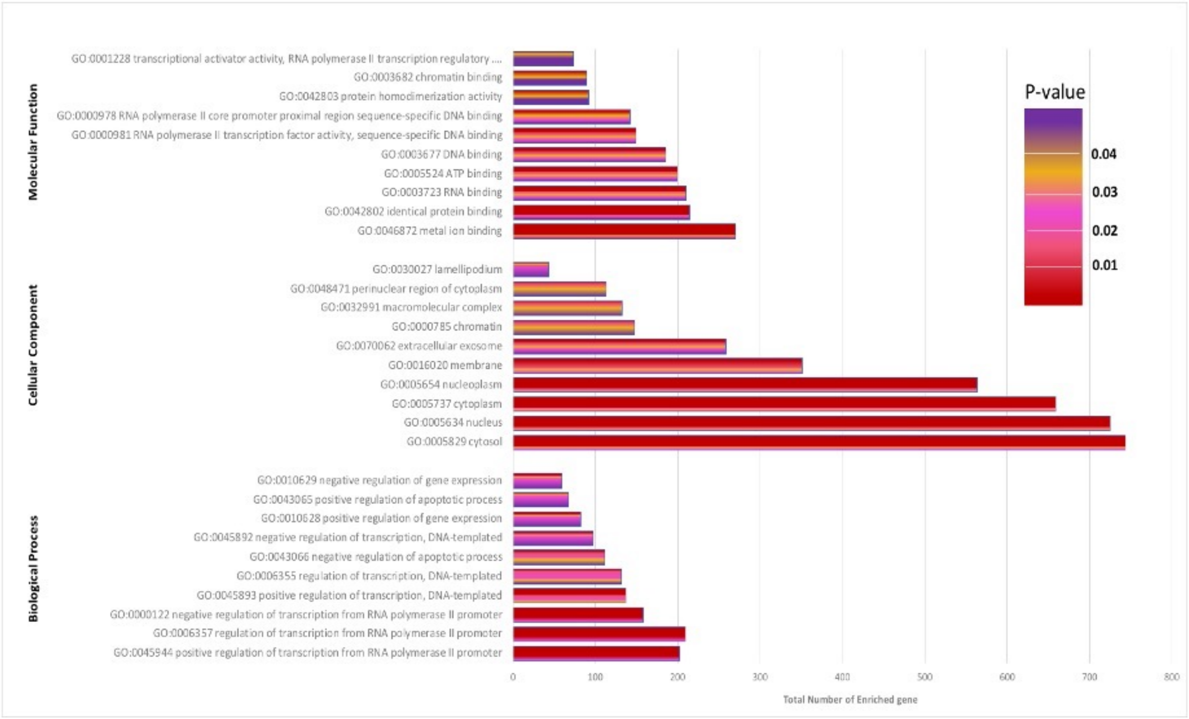

Figure S2. Represents functional enrichment analysis based on Gene Ontology analysis, revealing top ten highly enriched term of miRNA-target genes in Molecular Function, Biological Process and Cellular Component
